# Gesture recognition by instantaneous surface EMG images

**DOI:** 10.1038/srep36571

**Published:** 2016-11-15

**Authors:** Weidong Geng, Yu Du, Wenguang Jin, Wentao Wei, Yu Hu, Jiajun Li

**Affiliations:** 1 Zhejiang University, College of Computer Science, Hangzhou, 310027, China

## Abstract

Gesture recognition in non-intrusive muscle-computer interfaces is usually based on windowed descriptive and discriminatory surface electromyography (sEMG) features because the recorded amplitude of a myoelectric signal may rapidly fluctuate between voltages above and below zero. Here, we present that the patterns inside the instantaneous values of high-density sEMG enables gesture recognition to be performed merely with sEMG signals at a specific instant. We introduce the concept of an sEMG image spatially composed from high-density sEMG and verify our findings from a computational perspective with experiments on gesture recognition based on sEMG images with a classification scheme of a deep convolutional network. Without any windowed features, the resultant recognition accuracy of an 8-gesture within-subject test reached 89.3% on a single frame of sEMG image and reached 99.0% using simple majority voting over 40 frames with a 1,000 Hz sampling rate. Experiments on the recognition of 52 gestures of NinaPro database and 27 gestures of CSL-HDEMG database also validated that our approach outperforms state-of-the-arts methods. Our findings are a starting point for the development of more fluid and natural muscle-computer interfaces with very little observational latency. For example, active prostheses and exoskeletons based on high-density electrodes could be controlled with instantaneous responses.

A muscle-computer interface (MCI) is a communication system that transforms myoelectrical signals from mere reflections of muscle activities into interaction commands that convey the intent of the user s movement. A muscle is composed of many motor units (MU), and the “discharge” or “firing” of each MU activation generates a “motor unit action potential” (MUAP), which is the sum of the contributions from the individual fibres that compose the MU. Surface electromyography (sEMG) records a muscle’s electrical activity from the surface of the skin and thus reflects the generation and propagation of MUAPs. sEMG-based gesture recognition is the technical core of non-intrusive muscle-computer interfaces, which are often directed at controlling active prostheses^1^, wheelchairs^2^, exoskeletons^3^ or providing an alternative interaction method for video games^4^.

Gesture recognition based on sEMG can be naturally framed as a pattern classification problem in which a classifier is usually trained through supervised learning. It has generally been accepted that feeding the instantaneous value of myoelectric signals directly to a classifier is impractical and useless for pattern recognition techniques^5^,^6^. This belief is based on an empirical assumption that the instantaneous values of myoelectric signals are useless for gesture recognition because the raw myoelectric signal in each channel is non-stationary, non-linear, stochastic and unpredictable^7^,^8^,^9^. These features of the myoelectric signal reflect the constant variation of the actual set of recruited motor units within the range of available motor units and the arbitrary manner in which these motor unit action potentials superpose^10^. The amplitude of the myoelectric signal at any instant may rapidly fluctuate between voltages above and below zero^8^,^9^ and thus resembles a zero-mean random process whose standard deviation is proportional to the number of active motor units and the rate at which motor units are activated^11^. Therefore, existing gesture recognition methods using sEMG are largely based on a conventional pattern recognition algorithms (such as support vector machine^12^, hidden Markov model^13^, *etc.*) on sEMG feature space, i.e., the sequence of myoelectric signals of each channel often need to be transformed into a set of descriptive and discriminatory features extracted using a window of EMG data (or segment)^5^,^6^,^14^. Figure 1 shows a classical framework of gesture recognition using windowed sEMG. The optimal window length represents a compromise between classification error and controller delay in the field of assistive technology, physical rehabilitation and human computer interactions.

Existing gesture recognition approaches can be broadly divided into two categories: (1) methods based on sparse multi-channel sEMG^15^,^16^,^17^,^18^,^19^, and (2) methods based on high-density sEMG (HD-sEMG)^20^,^21^,^22^. Gesture recognition based on sparse multi-channel sEMG usually require precise positioning of the electrodes over the muscle^23^, thus limiting its use in MCI. HD-sEMG( i.e., sEMG recorded using two-dimensional electrode arrays) has enabled both temporal and spatial changes of the electrical potential to be recorded by multiple, closely spaced electrodes on the skin overlaying a muscle area^24^. Recently, more MCIs have been developed based on HD-sEMG^23^,^25^.

The collected HD-sEMG data are composed of myoelectric signals that characterize the spatiotemporal distribution of myoelectric activity over the muscles that reside within the electrode pick-up area. The collected HD-sEMG data also provide a global view of the varying states of electric fields on the surface of the involved muscles sampled via arrayed electrodes within the covered region, i.e., the instantaneous values of HD-sEMG present a relative global measure of the physiological processes underlying muscle activities at a specific time. However, whether the instantaneous HD-sEMG exhibits spatial patterns or is random like individual sEMG channels remains unclear.

We have determined that there are patterns inside the instantaneous HD-sEMG that are reproducible across trials of the same gesture and discriminative among different gestures. To verify this hypothesis computationally, we introduced the concept of an sEMG image and designed a series of experiments using three public databases employing an image-classification framework to recognize gestures from HD-sEMG.

An acquired HD-sEMG describes a potential distribution in space (as shown in Fig. 2), which yields the surface EMG image. The number of pixels (resolution) in sEMG images is defined by the array of electrodes, i.e., the number of electrodes and their inter-electrode distance (e.g., an electrode grid with 16 rows and 8 columns forms an sEMG image with 8 × 16 pixels). An instantaneous sEMG image is a single sample of a motor unit action potential distribution under an electrode grid at a specific time. The number of instantaneous sEMG images captured per second is the sampling frequency in time.

In general, the recognition of hand gestures by instantaneous sEMG images can be naturally framed as a problem of image classification, which can be solved by standard supervised learning: given a training set of captured instantaneous sEMG images labelled with performed hand gestures, we teach a classifier to predict the desired hand gesture for each incoming sEMG image. We adopted a deep-learning^26^,^27^,^28^,^29^ approach to solve the sEMG image-classification problem because its computational model based on deep convolutional neural networks (ConvNet) is trained end to end, from raw pixels to ultimate categories, without any additional information or manual design of feature extractors^30^. This approach thus fulfils our requirements for experimental verification.

We employed the deep-learning framework to recognize hand gestures from sEMG images and computationally elucidate the patterns in the instantaneous sEMG images (shown in Fig. 2). This process has two phases: an offline training phase and an online recognition phase. In the training phase, given the sEMG images and their gesture labels, an image classifier is trained to predict to which hand gesture an sEMG image belongs. In the recognition phase, the trained image classifier is utilized to recognize hand gestures from sEMG images.

A similar concept called sEMG topography^31^,^32^ or sEMG map^33^ was proposed for medical applications. Recently, sEMG map has also been utilized to recognize hand gestures^20^,^21^. Rojas *et al*.^20^ defined an sEMG map as a time-averaged 2D intensity map of sEMG signals, in which each pixel is the root mean square (RMS) value of a certain channel in a time window ( e.g., 3000 ms). The major difference between our instantaneous sEMG image and the sEMG map proposed by Rojas *et al*.^20^ is that our concept of instantaneous sEMG image is proposed to address per-frame gesture recognition. Only in the extreme case, when the window length of the sEMG map is reduced to one frame, the sEMG map is similar to our instantaneous sEMG image, except that our instantaneous sEMG image is directly formed from the raw sEMG signals while the sEMG map is the rectified sEMG signals ( i.e., the absolute values of the sEMG signals^10^). Experiment 3 indicated that our instantaneous sEMG image is superior to the sEMG map in terms of the gesture recognition accuracy.

## Experiments

### Experiment 1: Testing on an sEMG image and its sEMG difference image

**Figure 2:**
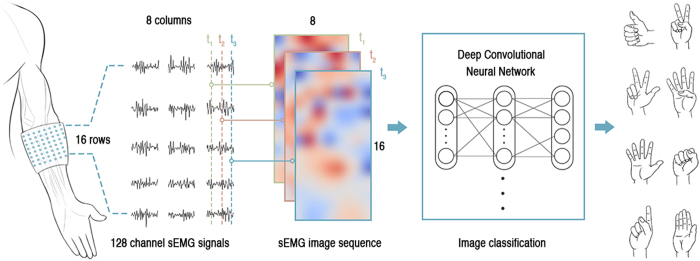
Schematic illustration of gesture recognition by instantaneous sEMG images.

To test this hypotheses, we newly proposed an experimental test-bed based on the framework of recognizing hand gestures by instantaneous sEMG image (shown in Fig. 2). The deep-learning framework is based on MxNet, a multi-language machine learning (ML) library to ease the development of ML algorithms, especially for deep-learning^34^. We also developed a non-invasive wearable device to collect HD-sEMG data for our verification experiments. This device consisted of 8 acquisition modules. Each acquisition module contained a matrix-type (2 × 8) electrode array with an inter-electrode horizontal distance of 7.5 mm and a vertical distance of 10.05 mm. The 128 sEMG signals were band-pass filtered at 20–380 Hz and sampled at 1,000 Hz with a 16-bit A/C conversion. The instantaneous values of sEMG signals at each sampling instant were arranged in a two-dimensional grid (in accordance with the electrode positioning). This grid was further converted to a grayscale image in which the units of the sEMG signal were converted from mV to colour intensity. The details of data preprocessing could be found in the methods section.

**Figure 3:**
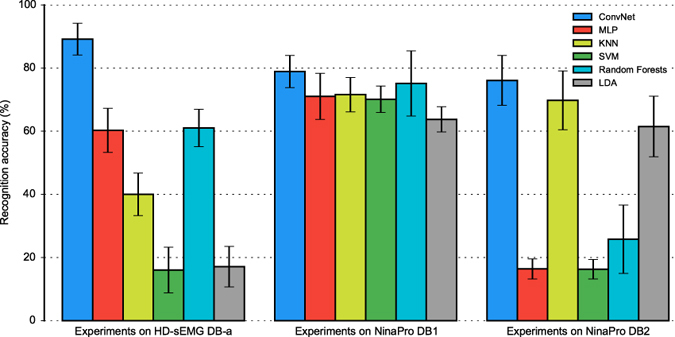
Recognition accuracy of 8 hand gestures with instantaneous values of sEMG signals for different datasets and recognition approaches. Each group of columns represents a specific experiment. Different colours represent different recognition approaches: ConvNet (deep convolutional neural network) with instantaneous sEMG images, MLP (multilayer perceptron), KNN (K-Nearest Neighbours), SVM (Support Vector Machine) Random Forests and LDA (Linear Discriminant Analysis). Error bars denote standard deviations.

**Figure 4:**
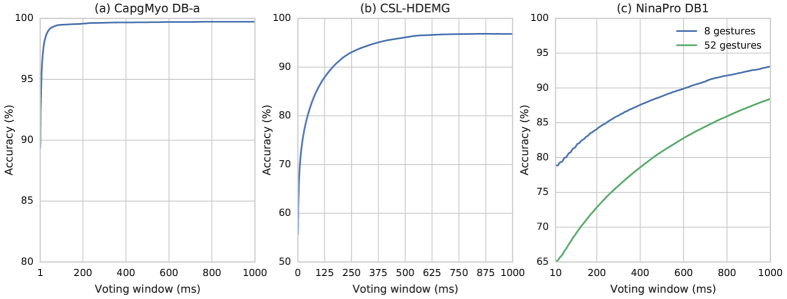
Recognition accuracies of hand gestures with different voting windows for three public databases. (**a**) Recognition accuracies of 8 finger gestures of CapgMyo DB-a. (**b**) Recognition accuracies of 27 finger gestures of CSL-HDEMG. (**c**) Recognition accuracies of 8 finger gestures and 52 hand gestures of NinaPro DB1.

In experiment 1, we recruited 18 healthy able-bodied subjects ranging in age from 23 to 26 years. Each subject was paid to perform 8 isometric and isotonic hand gestures identical to those in the NinaPro database^35^; each gesture was also recorded 10 times. We denote this dataset as DB-a in the following sections, which is a sub-database of our CapgMyo database. ConvNet was trained with the odd-numbered trials of each subject and tested with the remaining half. The results of this experiment, presented in Fig. 3, demonstrated that hand gestures can be recognized with an accuracy of 89.3% on a single instantaneous sEMG image. Higher recognition accuracies of 99.0% and 99.5% can be obtained by simple majority voting over the recognition results of 40 and 150 frames, respectively (Fig. 4(a)). At our sampling rate, 150 frames is equivalent to 150 ms, which is the window size suggested by several studies of pattern recognition based on prosthetic control^1^,^6^,^36^. The recognition accuracy was very high for instantaneous sEMG images, thus demonstrating that the patterns possibly exist in the HD-sEMG signals for discriminating hand gestures.

We also performed the test using the difference sEMG image ( i.e., changes between two consecutive sEMG images in the temporal sEMG sequence). The resulting recognition accuracy was 84.6% on a single difference image, and 99.4% using simple majority voting over 149 difference images. By simple majority voting over 150 instantaneous sEMG images together with their 149 difference images, we obtained a recognition accuracy of 99.6%. Such a high recognition accuracy on the difference sEMG images further verify that there are patterns in the instantaneous HD-sEMG signals from a temporal perspective.

Finally, we developed a prototype MCI based on real-time gesture recognition (shown in supplementary Figure S1), which recognizes 8 isometric and isotonic finger gestures (equivalent to Nos 13–20 in NinaPro^18^) using the 128 channels HD-sEMG signals recorded by our non-invasive wearable device. The HD-sEMG data from the 8 electrode arrays were packed in an ARM controller and transferred to a workstation via WIFI. Our MCI system displays the recognized hand gesture and the recorded HD-sEMG image in real-time. The deep learning classifier is running on a workstation with one NVidia Titan X GPU.

### Experiment 2: Testing with classical recognizers

Motivated by the very high accuracy of the HD-sEMG-based gesture recognition via deep learning, in the second experiment, we replaced ConvNet with conventional classifiers. The dataset was identical to that in experiment 1, DB-a, where instantaneous values of sEMG signals were directly formed as a feature vector (denoted as sEMG vector). This procedure ensured that the conventional classifier did not utilize any additional information or manually specified features of myoelectric activity residing within the electrode pick-up area. We evaluated five classical classifiers: MLP (multilayer perceptron), KNN (K-Nearest Neighbours), SVM (Support Vector Machine), Random Forests and LDA (Linear Discriminant Analysis). The resulting evaluation of the DB-a dataset demonstrated that patterns can be observed from gesture recognition with MLP and Random Forests. Thus, patterns can be captured by certain classical recognizers and not merely by the deep-learning framework.

### Experiment 3: Testing on the CSL-HDEMG dataset

We evaluated our ConvNet-based gesture recognition on the CSL-HDEMG database^23^, which contains HD-sEMG signals of 5 subjects performing 27 finger gestures, in which each subject recorded 5 sessions and performed 10 trials for each gesture in every session. The sEMG signals were bipolar recorded at a sampling rate of 2,048 Hz, by using an electrode array with 192 electrodes, covering the upper forearm muscles, forming a grid of 7 × 24 channels. We used the same evaluation procedure, i.e., the filtering and segmentation of the signals as well as the cross-validation scheme, as that in the experiments of Amma *et al*.^23^. For each recording session, we performed a leave-one-out cross-validation in which each of the 10 trials was used in turn as the test set and a classifier was trained by using the remaining 9 trials. Our method achieved an accuracy of 96.8% using simple majority voting over the entire segment of each trial, an 6.4% improvement over the latest work of gesture recognition base on HD-sEMG^23^. The recognition accuracies with different voting windows are presented in Fig. 4(b). The recognition accuracy reached 55.8% on a single frame of HD-sEMG signals, and it reached 89.3%, 90.4% and 95.0% using simple majority voting over 307, 350 and 758 frames, respectively, with a 2,048 Hz sampling rate.

We also evaluated the sEMG map proposed by Rojas *et al*.^20^, which is a time-averaged 2D intensity map of HD-sEMG signals. Using our ConvNet-based classifier and sEMG map with an averaging window of one frame, the recognition accuracy was 52.1%, which was 3.7% lower than using our instantaneous sEMG image. This indicates that our instantaneous sEMG image is superior to the sEMG map in terms of the gesture recognition accuracy.

### Experiment 4: Testing on the NinaPro dataset with sparse channels

In experiment 4, we performed the tests of 8 figure gestures on the NinaPro database^35^, a benchmark scientific database with ten electrodes on the forearm for hand prostheses. We performed this experiment because the patterns in the HD-sEMG data should also be embodied to a certain extent in the sEMG data. In the NinaPro dataset, 52 gestures were recorded 10 times by 27 subjects in sub-database 1 (DB1) and 50 gestures were recorded 6 times by 40 subjects in sub-database 2 (DB2). We transformed the instantaneous values of the sEMG signals at each instant into an image with 1 × 10 pixels, where the first eight components corresponded to the equally spaced electrodes around the forearm at the height of the radio-humeral joint and each of the last two components corresponded to electrodes placed on the main activity spots of the flexor digitorum superficialis and the extensor digitorum superficialis. Following the classification procedure of NinaPro^35^, for each sub-database of the NinaPro dataset, a ConvNet was trained with approximately two thirds of the trials of each subject and tested with the remaining one third. The accuracy was calculated as the proportion of correctly recognized images and averaged over all the subjects. For both NinaPro sub-databases, the recognition accuracy with instantaneous sEMG images was 78.9% for DB1 and 76.1% for DB2 (Fig. 3).

Moreover, we evaluated the ConvNet-based recognition of all 52 hand gestures of NinaPro DB1 using the aforementioned classification procedure. As shown in Fig. 4(c), the resulting recognition accuracy reached 65.1% on a single frame of sEMG signals, reached 75.3% using simple majority voting over 28 frames with a 100 Hz sampling rate and reached 96.7% using simple majority voting over the entire segment of each trial. With a preprocessing low-pass Butterworth filter (1st order, 1 Hz), Atzori et al.\cite{Atzori_SData_2014} achieved a recognition accuracy of 75.3% using a 200 ms window (20 frames), while our recognition accuracy reached 76.1% on single frame of sEMG signals and reached 77.8% using simple majority voting over a 200 ms window (20 frames). These results suggest that our approach based on sEMG image outperforms state-of-the-art methods for gesture recognition, even with the sEMG signals from sparse multiple channels. These results further confirmed our hypothesis that there are patterns inside the instantaneous sEMG data, even with the sEMG from sparse channels.

## Discussion

In this study, we performed a series of experiments to verify our assumptions on the patterns inside instantaneous sEMG images and demonstrate that the hand gestures of a specific subject will be effectively recognized directly on the instantaneous sEMG images with an image classifier trained on the samples of hand gestures from the same subject. We achieved state-of-the-art performance for sEMG-based gesture recognition on three public databases (NinaPro^35^, CSL-HDEMG^23^ and our CapgMyo). For 27 finger gestures from CSL-HDEMG, the recognition accuracy reached 55.8% on a single frame of HD-sEMG signals with a 2,048 Hz sampling rate, and it reached 96.8% using simple majority voting over the entire segment of each trial–an 6.3% improvement over the latest work^23^. For 52 hand gestures from NinaPro DB1, the recognition accuracy reached 65.1% on a single frame of sEMG signals with a 100 Hz sampling rate, and it reached 96.7% using simple majority voting over the entire segment of each trial. Using the same configuration^35^, we improved the resulting recognition accuracy with lower observational latency.

The HD-sEMG-based hand gesture recognition experiments revealed that electromyography data do contain patterns that are reproducible across trials of the same gesture and discriminative among different gestures for a group of individuals. Indeed, the question of “what are the essential components of the patterns”? remains open and must be answered by biologists and physiologists in the future. Furthermore, the instantaneous sEMG images also present a relative global measure of the underlying physiological processes of muscle activities at a specific time. This research will open new avenues for studying muscle characteristics and interpreting the physiological mechanisms of patterns in dynamic transitional motions via sEMG, in addition to static gestures.

From the perspective of interactions between humans and machines, our findings provide a starting point for developing more fluid and natural muscle-computer interfaces with very little observational latency, particularly for applications in neuromuscular diagnosis, assistive technology, physical rehabilitation and human-computer interactions.

Currently, our classification method is based on ConvNet, which is computational expensive. In the future work, we plan to investigate more sophisticated classification algorithms for sEMG-based gesture recognition.

## Methods

### Data and code availability

Our CapgMyo database and the codes are available at http://zju-capg.org/myo.

### Acquisition protocol

CapgMyo DB-a consisted of recordings of 8 finger gestures, and each gesture was held for 3 to 10 seconds, followed by 7 seconds of rest. The set of gestures was recorded 10 times by each subject. The 8 acquisition modules were wrapped around the right forearm and formed an 8 × 16 electrode array. Subjects sat comfortably in an office chair and rested their arms on a desktop. Before donning the devices, the skin of the forearms was cleaned with alcohol. The imitation stimulus was visual. The subjects were asked to mimic a video of hand gestures shown on the screen with their right hand. The video was then used to generate a label for each sample.

The study was conducted in accordance with the Declaration of Helsinki and approved by the Ethics Committee of Zhejiang University, China. Written informed consent was obtained from all subjects.

### Data preprocessing

For CapgMyo, power-line interference was removed. For CSL-HDEMG^23^, the data were band-pass filtered and segmented as that in the experiments of Amma *et al*.^23^. In our evaluation, the sEMG signals of CSL-HDEMG at each sampling instant were preprocessed by a 3 × 3 spatial median filter. Rectification is to estimate the amplitude of the sEMG signals by computing the absolute values of the sEMG signals^10^. The sEMG signals of CapgMyo and CSL-HDEMG were not rectified. The sEMG signals of NinaPro DB1^35^ have been rectified and smoothed by the acquisition device. For NinaPro DB2, the sEMG signals were downsampled to 100 frames per second, which was the sampling rate of DB1, and preprocessed similar to that used for DB1.

### Instantaneous sEMG image

The instantaneous values of HD-sEMG signals at each sampling instant were arranged in a two-dimensional grid in accordance with the electrode positioning. This grid was further converted to a grayscale image with a linear transform in which the units of the sEMG signal were converted from mV to colour intensity. For our CapgMyo database, an instantaneous sEMG image was formed as an 8 × 16 grayscale image by linearly transforming the values of sEMG signals from [−2.5 mV, 2.5 mV] to [0, 1].

### ConvNet architecture

Our ConvNet had eight layers. The input to the ConvNet consisted of a 8 × 16 image for CapgMyo, a 7 × 24 image for CSL-HDEMG^23^ and a 1 × 10 image for NinaPro^35^. The first two hidden layers were convolutional layers, each of which consisted of 64 filters of 3 × 3 with stride 1. The next two hidden layers were locally connected^37^, each of which consisted of 64 non-overlapping filters of 1 × 1. The next three hidden layers were fully connected and consisted of 512, 512 and 128 units, respectively. The network ended with an *G*-way fully connected layer and a softmax function, where *G* is the number of gestures to be classified. We adopted (1) ReLU non-linearity^28^ after each hidden layer, (2) batch normalization^38^ after the input and before each non-linearity, and (3) dropout^39^ with a probability of 0.5 on the fourth, fifth and sixth layers.

### ConvNet training

We used stochastic gradient descent (SGD)^40^ with a data batch size of 1,000, an epoch number of 28, and a weight decay of 0.0001 in all experiments. The learning rate started from 0.1 and was divided by 10 after the 16th and the 24th epochs, The weights of the ConvNet were initialized as described in a previous study^41^. In order to prevent an overfitting of the small training set in experiments 1, 3 and 4, the ConvNet was initialized by pre-training on the union of the training sets of all subjects in each round. In experiment 3, the statistics of the batch normalization layers, i.e., mean and variance of each input channel, were re-calculated with the test data.

### Conventional classifiers

For MLP, we used a single hidden layer of 1,024 units with ReLU non-linearity and applied the same training scheme as that of the ConvNet case. For KNN, SVM, Random Forests and LDA, we used the implementation and default hyper-parameters of Scikit-learn 0.17.0^42^. The training set was downsampled by a factor of 9 in experiment 2 for computational ease.

### Hyper-parameter tuning

The hyper-parameters of the entire recognition model included 1) a method for detecting malfunctioning channels; 2) a method for the removal of power-line interference; 3) a method for rectification; 4) a method for low-pass filtering; 5) a method for amplitude normalization; 6) a method for cross-talk removal; 7) a colour scheme for instantaneous sEMG images; and 8) a method for training data augmentation.

The hyper-parameters were selected by comparing the recognition accuracy of a specific configuration with that of a baseline model on DB-a (as shown in Table 1). We used the data of first 9 subjects of DB-a for hyper-parameter tuning. The model was trained with the odd numbered trials and tested with the even numbered trials. The hyper-parameter configurations are provided in Table 1.

The result, as presented in Table 1, demonstrated that only the removal of power-line interference increased the recognition accuracy. Although low-pass filtering at 75 Hz improved the recognition accuracy, it introduced a latency of 3 ms^43^, equivalent to the average latency introduced by an analysis window of 6 ms^6^. With majority voting on the recognition result in 6 ms, the recognition accuracy of the baseline configuration was 92.5%, which are significantly higher than the accuracy obtained by low-pass filtering. This suggests that the “noise” removed by low-pass filtering contained useful patterns.

## Additional Information

**How to cite this article**: Geng, W. *et al*. Gesture recognition by instantaneous surface EMG images. *Sci. Rep.* **6**, 36571; doi: 10.1038/srep36571 (2016).

**Publisher’s note**: Springer Nature remains neutral with regard to jurisdictional claims in published maps and institutional affiliations.

## Supplementary Material

Supplementary Movie

Supplementary Information

## Figures and Tables

**Figure 1:**
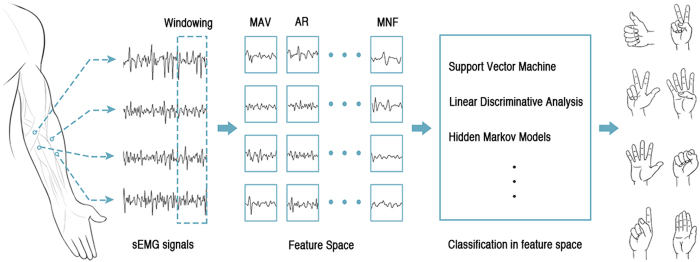
Schematic illustration of gesture recognition by windowing sEMG signals. MAV: mean absolute value. AR: auto-regressive coefficients. MNF: mean frequency^44^.

**Table 1:** Hyper-parameter tuning on DB-a.

Hyper-parameter configuration	Accuracy	Description
Baseline	85.5%	Without any preprocessing or data augmentation. Create a RGB image by replicating grayscale sEMG image three times. Use the same ConvNet architecture as that in experiment 1, with the exception that the last two convolutional layers are removed. The recognition accuracy reached 92.5% using simple majority voting over 6 frames.
Malfunctioning channels corrected	85.4%	Detect and correct malfunctioning channels as described in a previous study^45^.
Power-line interference removed	86.4%	Band-stop filtered between 45 and 55 Hz using a second-order Butterworth filter to remove power-line interference.
Full-wave rectified	81.2%	Full-wave rectification followed by low-pass filtering is a standard amplitude estimation technique^46^. Full-wave rectification takes the absolute value of the sEMG signals.
Low-pass filtered	88.2%	Full-wave rectified and low-pass filtered using a second-order Butterworth filter with a cut-off frequency of 75 Hz.
Amplitude normalized	85.0%	sEMG signals are normalized by maximum voluntary contraction (MVC), which is a technique used to reduce the unwanted variability by dividing the sEMG signals by a reference value^10^. For each subject, the reference value is computed as the maximum of the full-wave rectified and low-pass filtered (3 Hz, second-order Butterworth) max-force data which is collected during the same acquisition session with eight gestures.
Cross-talk removed	80.0%	sEMG signals are preprocessed by independent component analysis (ICA), which has been found to be successful for cross-talk removal^47^.
Jet colour scheme	83.7%	Create a RGB image from grayscale sEMG image by Jet colour scheme^48^.
Training data augmented	84.2%	Augment training data by circularly translate ±1 pixels in row direction to simulate electrode shift^49^.

Each configuration is a variation of the baseline with respect to one hyper-parameter.
