# Supplementary material for: Gesture recognition by instantaneous surface EMG images
Source: Sci Rep. 2016 Nov 15;6:36571. doi: 10.1038/srep36571 (PMC5109222; doi:10.1038/srep36571)
Supplement: Supplementary Information [file srep36571-s2.pdf]

Supplementary Information

Gesture recognition by instantaneous surface EMG images

Weidong Geng[1,*], Yu Du[1], Wenguang Jin[1], Wentao Wei[1], Yu Hu[1], and Jiajun Li[1]

[1]Zhejiang University, College of Computer Science, Hangzhou, 310027, China
[*]gengwd@zju.edu.cn

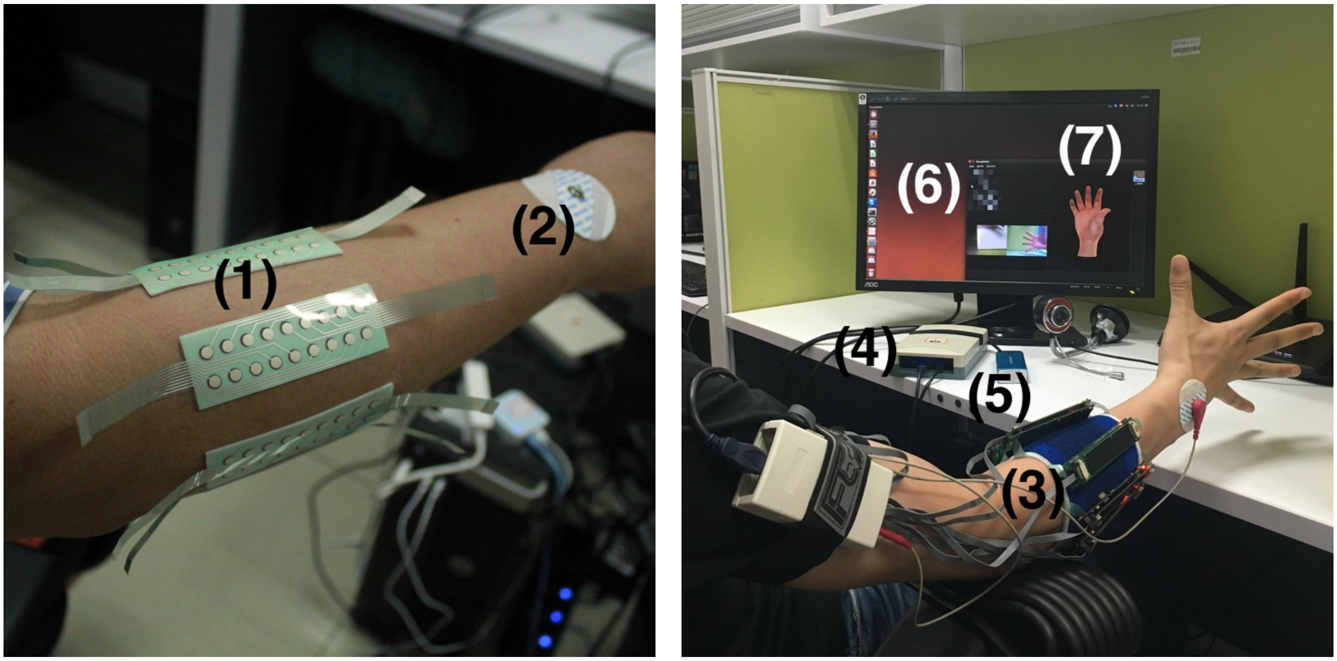

Figure S1: The MCI system: (1) electrode array; (2) reference electrode; (3) acquisition modules; (4) data transmission module; (5) lithium battery; (6) instantaneous sEMG image; (7) recognized gesture.

Video S1: Real-time gesture recognition by the proposed method.
